# Relative Influence of Socioeconomic, Psychological and Sensory Characteristics, Physical Activity and Diet on 5-Year Weight Gain in French Adults

**DOI:** 10.3390/nu9111179

**Published:** 2017-10-28

**Authors:** Aurélie Lampuré, Katia Castetbon, Mohamed Hanafi, Amélie Deglaire, Pascal Schlich, Sandrine Péneau, Serge Hercberg, Caroline Méjean

**Affiliations:** 1 Centre de Recherche en Épidémiologie et Statistiques, Équipe de Recherche en Épidémiologie Nutritionnelle, Université Paris 13, Inserm (U1153), Inra (U1125), Cnam, COMUE Sorbonne Paris Cité, F-93017 Bobigny, France; s.peneau@eren.smbh.univ-paris13.fr (S.P.); s.hercberg@eren.smbh.univ-paris13.fr (S.H.); caroline.mejean@inra.fr (C.M.); 2 Centre de Recherche en Épidémiologie, École de Santé Publique, Biostatistiques et Recherche Clinique, Université Libre de Bruxelles, B-1070 Bruxelles, Belgium; katia.castetbon@ulb.ac.be; 3 École Nationale Vétérinaire, Agroalimentaire et de l’Alimentation, ONIRIS, Unité de Sensométrie et de Chimiométrie, site de la Géraudière, BP82225, 44322 Nantes CEDEX 3, France; mohamed.hanafi@oniris-nantes.fr; 4 Unité Inra STLO Science et Technologie du Lait et de l’Œuf, UMR 1253, 35042 Rennes, France; amelie.deglaire@agrocampus-ouest.fr; 5 Centre des Sciences du Goût et de l’Alimentation, UMR 6265 CNRS, UMR 1324 Inra, 21000 Dijon, France; pascal.schlich@dijon.inra.fr; 6 Département de Santé Publique, Hôpital Avicenne (AP-HP), F-93017 Bobigny, France; 7 INRA, UMR 1110 Marchés, organisations, institutions et stratégies d’acteurs (MOISA), F-34000 Montpellier, France

**Keywords:** weight gain, dietary intake, individual characteristic, physical activity, determinant, structural equation modelling

## Abstract

Individual characteristics, dietary intake and physical activity influence weight status; however, the contribution of each factor to weight change has not been studied. The objective was to confirm a conceptual framework by simultaneously assessing the relative influence of socioeconomic, psychological and sensory characteristics, physical activity, and dietary intake on five-year weight gain in French adults. Individual characteristics, physical activity, and dietary data were assessed at baseline in 8014 participants in the NutriNet-Santé cohort. Self-reported anthropometric data were collected at baseline and five years later. Structural equation models, stratified by baseline body mass index (BMI), were used to perform analyses. Dietary restraint was a direct predictor of weight gain, with a stronger effect than age or intake of energy-dense foods, both in non-overweight and overweight participants. In non-overweight individuals only, intake of nutrient-dense foods and physical activity were inversely associated with weight gain. Regarding dietary intake, fat liking was the most important predictor of nutrient-dense food intake and was also related to energy-dense food intake. In these models, dietary restraint appears to be a direct predictor of weight gain and fat liking is a strong determinant of dietary intake. The influence of dietary restraint on weight gain, not explained by diet, warrants further investigation.

## 1. Introduction

Obesity is a worldwide public health concern [1,2], and the potential role of energy-dense diets, as well as the beneficial effects of nutrient-dense foods and physical activity, have been demonstrated [1,3,4,5,6,7]. Weight change is, therefore, influenced by dietary intake and physical activity [8,9,10,11,12,13], but may also be affected by more distant determinants interacting with each other [8,14,15,16,17].

Individual sensory liking, such as liking for fat sensation, sweet and salty tastes, may be associated with body weight. Indeed, previous studies have shown a positive association between liking for fat sensation and body mass index (BMI) [18,19] and an increased risk of obesity [20], and this relationship was largely mediated by food intake. Indeed, subjects with high fat liking have higher fat intake and lower intake of nutrient-dense foods [21]. Regarding psychological characteristics such as cognitive restraint, studies have shown a positive association with BMI [22,23], but the relationship with weight gain is controversial [15,22,23], whereas recent dieting (i.e., to be on a diet plan recently) seems to predict subsequent weight gain [15,23]. Uncontrolled eating and emotional eating also appear to be associated with higher BMI [23,24]. Dietary intake may also explain these relationships; cognitive restraint is associated with higher vegetable intake and lower intake of French fries [25], whereas uncontrolled and emotional eating are associated with higher consumption of energy-dense foods [25,26]. More distant determinants, such as demographic characteristics (sex, age) and socioeconomic status might also influence weight status [17,27,28] through dietary intake. For example, higher socioeconomic status is associated with a better diet quality [29,30,31].

Although various determinants of weight change have already been identified in the literature, very few studies considered simultaneously a large set of determinants of weight status. In addition, the relative influence of each direct and distant determinant of weight status, compared to others, and their causal paths and complex interactions have not been considered to date. In order to study the complex hierarchical framework of the effect of determinants on weight change, we constructed a theoretical model based on the literature.

The aim of the present study was to test an a priori conceptual framework by simultaneously assessing the relative contribution of sensory, psychological and socio-economic characteristics, physical activity and dietary intake on five-year weight change in French adults. In addition, the relative effects of these individual characteristics on dietary intake were evaluated.

## 2. Materials and Methods

### 2.1. Study Population

We used data from the NutriNet-Santé study, a large web-based observational cohort launched in France in 2009 with a scheduled follow-up of 10 years. It was implemented in a general population and targeted internet-using adult volunteers. Briefly, eligible participants were recruited by a variety of means. Initially a vast multimedia campaign (television, radio, national and regional newspapers, posters, and internet) called for volunteers and provided details on the study’s specific website (http://www.etude-nutrinet-sante.fr). Then, multimedia campaigns were repeated every six months. Further information is maintained on a large number of websites (national institutions, city councils, private firms, web organizations). A billboard advertising campaign is regularly updated via professional channels (e.g., doctors, pharmacists, dentists, business partners, municipalities). The study was designed to investigate the relationship between nutrition and health, as well as determinants of dietary behaviour and nutritional status [32]. In order to be included in the cohort, participants had to fill out an initial set of questionnaires assessing dietary intake, physical activity, anthropometry, lifestyle, socio-economic conditions, and health status. As part of their follow-up, participants complete the same set of questionnaires every year. Moreover, each month, they are invited to fill out complementary questionnaires related to determinants of dietary behaviour, nutritional and health status. All questionnaires are completed online via the NutriNet-Santé website.

This study was conducted according to guidelines laid down in the Declaration of Helsinki, and all procedures were approved by the Institutional Review Board of the French Institute for Health and Medical Research (IRB Inserm No. 0000388FWA00005831) and the “Commission Nationale Informatique et Libertés” (CNIL No. 908450 and No. 909216). Electronic informed consent was obtained from all subjects. This study is registered in EudraCT (No. 2013-000929-31).

### 2.2. Data Collection

This longitudinal analysis focused on participants included in the NutriNet-Santé study between May 2009 and May 2010. Data regarding age, sex, socioeconomic status, smoking, sensory and psychological factors, physical activity, and dietary intake used in this analysis were collected in 2010. Weight and height data were collected in 2010 and five years later in 2015.

#### 2.2.1. Assessment of Weight and BMI

Height and weight data were collected at baseline and each year thereafter by a self-administered anthropometric questionnaire [33]. BMI was calculated as the ratio of weight to the square of height. Participants with BMI < 25 kg/m^2^ at baseline were classified as non-overweight (including 332 underweight participants in our study) and participants with BMI ≥ 25 kg/m^2^ were considered overweight (including obese) in accordance with WHO reference values [34]. The relative weight change over five years was calculated using five-year weight data subtracting the baseline weight data, divided by baseline weight data.

#### 2.2.2. Assessment of Dietary Intake

At enrolment, and each year thereafter, participants were invited to provide three random 24 h dietary records during a two-week period (one weekend day and two weekdays) [33]. The dietary record is completed via an interactive interface and is designed for self-administration on the internet. The web-based dietary assessment method relies on a meal-based approach, recording all foods and beverages (type and quantity) consumed at breakfast, lunch, dinner, and all other eating occasions. The accuracy of web-based 24 h dietary records has been assessed by comparing them to interviews by trained dieticians [33] and against 24 h urinary biomarkers [35,36]. Foods were first classified according to information provided in the French National Nutrition and Health Program guides [37]. For our purpose, several food groups were merged and formed: fruits and vegetables; meat and processed meat; fish; starchy foods; whole grain products; cheese; milk and yogurt; salted snacks, appetizers and savoury sauces; oil; butter and other added fats; fatty-sweet products; sweetened beverages; and alcoholic beverages. For each participant, daily average quantity of each food group (in grams/day) was calculated from 24 h records, including weighting according to the day of the week (weekdays or weekend). Diet-underreporting participants were identified by the method proposed by Black [38] and were excluded from analysis since this phenomenon is strongly linked to weight status [39]. However, participants had the option of indicating whether the reported consumption were representative of their usual diet or considerably differed (due to dieting for example, illness, a social event, etc.) and that information was taken into account to identify specific conditions that could objectively explain low energy intake. Thus, individuals which declare to be on a diet plan were not considered as under-reporters and were not excluded of analysis.

#### 2.2.3. Assessment of Physical Activity

Physical activity level was assessed using the French short version of the International Physical Activity Questionnaire [40]. Weekly energy expenditure expressed in Metabolic Equivalent Task -minutes/week (MET­-minutes/week) was estimated and three levels of physical activity were defined: low (<30 min/day), moderate (30–60 min/day), and high (≥60 min/day).

#### 2.2.4. Assessment of Liking for Fat-and-Salt and Fat-and-Sweet

Liking for fat-and-salt and fat-and-sweet sensations was assessed using the PrefQuest [41] in 2010. This questionnaire also assesses liking for salty and sweet tastes. Briefly, 83 relevant items were divided into liking for salt (11 items) and sweet (21 items) tastes, fat-and-salt (31 items) and fat-and-sweet (20 items) sensations. The questionnaire included four types of items: (i) liking for sweet, fatty-sweet, and fatty-salty foods; (ii) preferred level of salt, sweets, fat-and-salt, or fat-and-sweet seasoning; (iii) preferred drinks (sweet/sweetened or unsweetened) on a restaurant menu; and (iv) dietary behaviour in terms of sweet, salty, and fatty foods. Liking scores for fat-and-salt and fat-and-sweet sensations were computed as detailed previously [41,42] and range from 0 to 10.

#### 2.2.5. Assessment of Psychological Characteristics

Psychological data were collected in 2010 using the Three-Factor Eating Questionnaire, which is a self-assessment instrument of eating behaviour (TFEQ-R21) [43]. It covers three eating behaviour domains: the cognitive restraint scale (six items) assesses control over food intake to influence body weight; the emotional eating scale (six items) measures the propensity to overeat in relation to negative mood states; and the uncontrolled eating scale (nine items) assess the tendency to lose control over eating when feeling hungry or when exposed to external stimuli. The three scales range from 0 to 10 [44].

#### 2.2.6. Assessment of Socio-Demographic, Economic and Lifestyle Characteristics

Socio-demographic (age and sex: women and men), economic (education: primary, secondary, undergraduate or postgraduate; household income per consumption unit), and lifestyle data (smoking status: never, former, or current smoker; history of dieting: never, former, or current dieter) were collected in 2010.

### 2.3. Statistical Analyses

**Figure 1:**
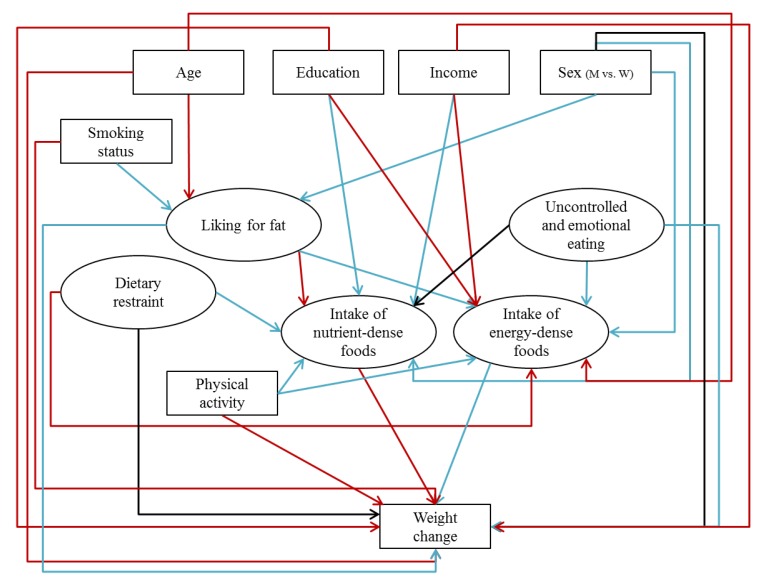
Theoretical model, based on the literature of the association between individual characteristics, dietary intake, and weight change (latent variables are presented in ovals and observed variables are presented in rectangles). Red arrows represent potential inverse associations, blue arrows represent potential positive associations, and black arrows represent controversial or not well-known associations. M: men, W: women.

Our analysis focused on participants included in the NutriNet-Santé study between May 2009 and May 2010 living in a French metropolitan area, who had self-reported height and weight data at baseline and five years later, who had completed three 24 h dietary records at baseline, who were not energy under-reporters, and who had no missing data for age, sex, smoking, dieting, physical activity, socioeconomic, sensory and psychological factors. Comparisons between baseline and five years later data showed slight changes in physical activity and dietary intake, highlighting that behaviours were unchanging during the five years (Supplemental Table S1). Description and comparison of participants at baseline were performed using Student’s *t*-test and the chi-square test, as appropriate. Correlations between individual characteristics were also investigated using Pearson and Spearman correlations, as appropriate (Supplemental Table S2). Globally, the majority of correlations were significant and expected correlations, such as between age and income, or educational level and smoking, were found, as well as between income and education, for example. Regarding food groups, correlations were also mostly significant (data not tabulated). The associations between weight change and its determinants were estimated using a structural equation modelling (SEM) approach to test the theoretical model describing their relationships (Figure 1). The SEM approach is a multidimensional statistical method that represents a confirmatory approach to multivariate analysis and allows considering several exposures and outcomes at once. It overcomes the limit of classic regression models allowing to simultaneously test several relationships among multiple independent and dependent variables in one model [45]. The main hypothesis was that individual factors may indirectly influence weight change through dietary intake, and may also have a direct effect on weight change.

#### 2.3.1. Theoretical Construct

In a first step, we have elaborated a theoretical model summarizing associations identified in the literature between individual characteristics, dietary intake, and weight status (Figure 1). Part of the cited studies were conducted on the NutriNet-Santé cohort and others were meta-analyses and literature reviews. When the relationship has not been investigated in reviews or in our cohort, we cited the most recent studies.

#### 2.3.2. Development of the Measurement Model

In a second step, we constructed the measurement model by specifying the latent variables formed by measured variables using confirmatory factor analysis. Thus, we grouped highly-correlated manifest variables into latent factors as follows: (i) “liking for fat” measured by indicator variables from the PrefQuest questionnaire, liking for fat-and-salt and liking for fat-and-sweet scores; (ii) “intake of nutrient-dense foods” assessed by the intake of fruits and vegetables, fish, milk and yogurt, whole grain products, and oil; (iii) “intake of energy-dense foods” measured by the intake of starchy foods, meat and processed meat, cheese, salted snacks, appetizers and savory sauces, butter and other added animal fats (cream and lard), fatty-sweet products, sweetened soft drinks (including artificially sweetened drinks), and alcoholic beverages; (iv) “uncontrolled and emotional eating” was assessed by uncontrolled eating and emotional eating scores; and (v) “dietary restraint” was measured by cognitive restraint score and history of dieting.

#### 2.3.3. Development of the Structural Model

In a third step, the structural equation modelling approach called LISREL (LInear Structural RELations) [46] was used to confirm the full model. It was composed of the measurement model (latent concepts formed by manifest variables) and the structural model (associations between latent variables). The outcome variable was the relative five-year weight change. Independent variables which contribute to weight change, but were not part of a latent concept, such as age, sex, education, household income, smoking status, and physical activity were also included in the full model. 

#### 2.3.4. Assessment of the Validity

Three fit indexes were used to judge the fit of the model: the standardized root mean square residual (SRMR), the root mean square error of approximation (RMSEA), and the comparative fit index (CFI) [47]. To indicate a good fit, cut-offs are SRMR < 0.1, RMSEA ≤ 0.05, or at least ≤0.08, and CFI ≥ 0.90. In the final SEM models, only significant associations remained (*p* < 0.05). Standardized parameter estimates were presented to remove scaling effects and to compare parameters in models. They can be interpreted as beta weights in a multiple regression. Standardized residuals were examined to determine if conceptually-appropriate changes could be made to improve model fit (e.g., patterns of residual correlations). Structural equation modelling was conducted with R v3.1.2 (www.cran.r-project.org), using the package “lavaan” [48]. The robust maximum likelihood estimation (ML) method was used and the significance level was set at *p* < 0.05.

## 3. Results

### 3.1. Description of the Population

Among the 49,818 participants included in the NutriNet-Santé study in May 2010, 31,407 had available data for height and weight at baseline. We excluded 2961 participants with fewer than three 24 h dietary records or identified as diet under-reporters. Next, 3578 participants with missing data for sensory liking and 1291 individuals with missing data for psychological characteristics at baseline were also excluded. Furthermore, 3704 participants with missing data for physical activity and 1871 subjects with missing data for education or income per consumption unit were excluded. Finally, 9988 participants with missing data for weight five years later were excluded, which left 8014 participants available for analysis (5730 women and 2284 men). Compared with excluded subjects (excluded participants with available anthropometric data at baseline and at five years (Supplemental Table S3) and all excluded individuals (Supplemental Table S4)), individuals included in our analysis were older and had higher monthly income, the percentages of men and of those with high education were higher and the proportion of smokers was lower.

First, we tested the theoretical model (Figure 1), including BMI at baseline, since its influence on weight change may be strong [49]. Results showed that BMI at baseline was the main predictor of weight change (data not shown), and it concealed associations between individual factors, dietary intake, physical activity, and weight change. We, therefore, elaborated stratified models by BMI at baseline: a model for non-overweight persons (BMI < 25 kg/m^2^) and a model for overweight participants, including obese persons (BMI ≥ 25 kg/m^2^), to investigate the contribution of each determinant to weight change.

Compared to overweight subjects (Table 1), percentages of women, postgraduates, never-smokers, and never-dieters were higher in non-overweight participants. Mean age and mean BMI were higher in overweight subjects than in non-overweight individuals, whereas mean income was lower. Moreover, scores of liking for fat-and-salt, cognitive restraint, uncontrolled eating and emotional eating were lower in non-overweight subjects. In addition, since relative weight change between initial weight and weight five years later was positive, on average, participants gained weight. Regarding dietary intake, non-overweight subjects had lower intakes of energy, meat and processed meat, fish, starchy foods, cheese, milk and yogurt, salted snacks, appetizers and sauces, sweetened soft drinks, and alcoholic beverages, whereas they had higher intakes of fruits and vegetables, whole grain products, and sugar and sugary products, compared to overweight participants.

### 3.2. Structural Equation Modeling

The fit statistics were, in the model with non-overweight individuals: CFI = 0.91, RMSEA = 0.049 (0.047–0.051), and SRMR = 0.036; and in the model with overweight subjects: CFI = 0.87, RMSEA = 0.053 (0.050–0.056) and SRMR = 0.046.

**Figure 2:**
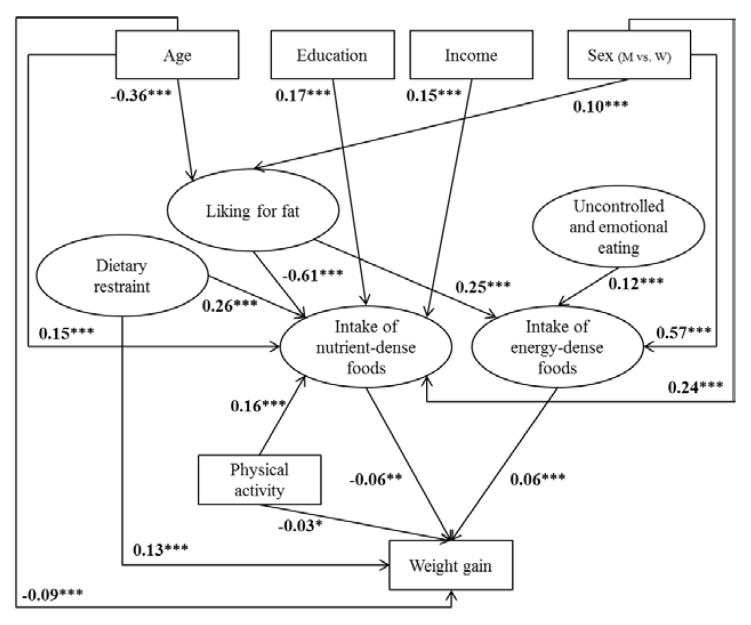
Determinants related to five-year weight gain in non-overweight participants (*n* = 5546). Path coefficients in the model can be interpreted as standardized regression weights. Latent variables are presented in ovals and observed variables are presented in rectangles. Fit indices for this model were standardized root mean square residual (SRMSR) = 0.036, root mean square error of approximation (RMSEA) = 0.049 (0.047–0.051), and comparative fit index (CFI) = 0.91. Significance values: * *p* < 0.05, ** *p* < 0.01, *** *p* < 0.0001. M: men, W: women.

**Figure 3:**
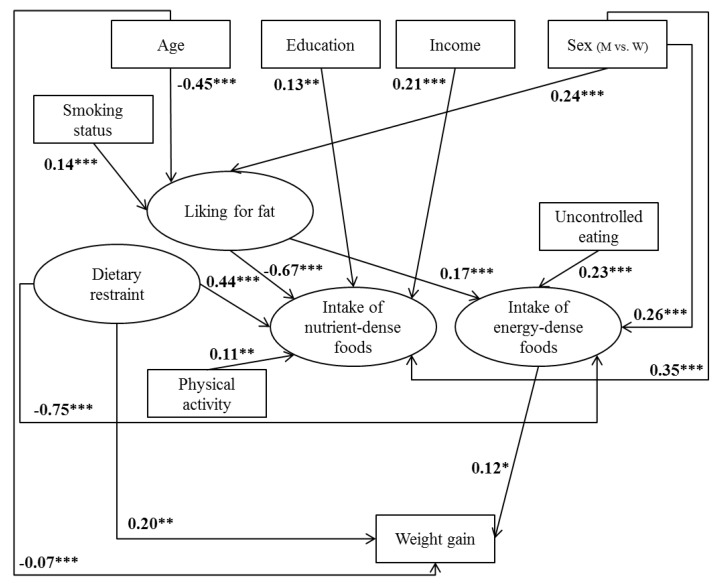
Determinants related to five-year weight gain in overweight and obese participants (*n* = 2468). Path coefficients in the model can be interpreted as standardized regression weights. Latent variables are presented in ovals and observed variables are presented in rectangles. The emotional eating variable did not fit into this model; only the measured variable “uncontrolled eating” was considered. Fit indices for this model were standardized root mean square residual (SRMSR) = 0.046, root mean square error of approximation (RMSEA) = 0.053 (0.050–0.056), and comparative fit index (CFI) = 0.87. Significance values: * *p* < 0.05, ** *p* < 0.01, *** *p* < 0.0001. M: men, W: women.

Figure 2 and Figure 3 illustrate structural equation models with arrows representing statistically significant associations with standardized parameter estimates. In non-overweight (Figure 2) and overweight (Figure 3) participants, men had a higher liking for fat and higher intake of both nutrient-dense and energy-dense foods than women. In addition, age was inversely related to fat liking, and fat liking was positively related to intake of energy-dense foods and inversely associated with intake of nutrient-dense foods, with the highest estimates. High dietary restraint was associated with high intake of nutrient-dense foods, whereas high uncontrolled (in overweight participants) and emotional eating was associated with greater intake of energy-dense foods. In contrast, uncontrolled and emotional eating was not associated with intake of nutrient-dense foods, nor with weight gain. In both samples, education, income, and physical activity levels were positively associated with intake of nutrient-dense foods, but no such association was found with intake of energy-dense foods. Regarding a direct influence on weight gain, age was inversely related to weight gain in both samples, while dietary restraint and intake of energy-dense foods were both positively associated with weight gain. In contrast, sex, education, income, smoking, and uncontrolled and emotional eating were not significantly associated with weight gain in either sample.

Different pathways were also found between models in non-overweight and overweight individuals. Indeed, in non-overweight participants, intake of nutrient-dense foods and physical activity were inversely associated with weight gain. In addition, age was positively associated with intake of nutrient-dense foods, but was not associated with intake of energy-dense foods in either sample. In overweight participants, smokers and former smokers had higher liking for fat than never-smokers, and dietary restraint was also inversely related to intake of energy-dense foods, with a high estimate.

## 4. Discussion

To our knowledge, this study, using structural equation modeling, was the first to simultaneously investigate the relative influence of direct predictors of weight gain and more distant determinants that probably influence weight status through dietary intake. In non-overweight and overweight participants, dietary restraint was the main predictor of weight gain, followed by age and intake of energy-dense foods, according to loadings. In non-overweight individuals, intake of nutrient-dense food and physical activity were inversely associated with weight gain. Regarding indirect determinants that may have an effect through dietary intake, fat liking and sex appear to be the strongest predictors of dietary intake. In addition, high uncontrolled eating was associated with higher intake of energy-dense foods, while higher socioeconomic status, high dietary restraint, and high physical activity were associated with higher intake of nutrient-dense foods.

Dietary restraint had a direct influence on weight gain in both samples, and was the most important predictor, followed by other common determinants, such as dietary intake, physical activity, or age. This original finding may be explained by the co-occurrence of uninhibited and restrained eating patterns at an individual level [50], such as impulsivity [51] and disordered eating [52], that cannot be evaluated via cross-sectional dietary assessment. In addition, we assume that episodes of cognitive restraint and impulsivity might cause weight fluctuations and weight gain over the long-term [52,53]. This result is also in concordance with a study showing that recent dieting may be a proxy of susceptibility to weight gain [15].

As found in previous works [8,10], both intake of energy-dense foods and nutrient-dense foods appear to be direct determinants of weight gain, with opposing effects in non-overweight participants. In overweight and obese individuals, only intake of energy-dense foods was associated with weight gain. As they were more often on a diet, they probably modified their eating habits which may not be captured by cross-sectional dietary assessment and could explain the absence of relationship between intake of nutrient-dense food and weight gain. In non-overweight participants only, physical activity was inversely associated with weight gain, in agreement with previous studies [8,12,54] suggesting that physical activity may protect against weight gain. However, in overweight and obese individuals, descriptive analyses showed no difference in mean weight gain according to physical activity (data not shown). Finally, age was inversely associated with weight gain. Descriptive analyses showed that younger subjects gained more weight over five years than older participants (data not shown). This direct effect of age on weight gain may be explained by physiological changes [55].

Other individual characteristics included in the theoretical model were not directly associated with weight gain, highlighting their indirect influence through dietary intake. First, fat liking was the main predictor of dietary intake, followed by socioeconomic status and uncontrolled and emotional eating, well-known determinants of dietary intake. This original finding confirms that sensory liking is the first criterion in food choice [56]. Sensory fat liking was strongly associated with dietary intake, in particular with intake of nutrient-dense foods, in line with previous works [21,57,58]. We assume that individuals with increased liking for fat may consume less healthy foods because they find them less tasty; consequently, they may tend to replace them by their energy-dense variants [21].

A socio-economic gradient, through education and income, was observed for intake of nutrient-dense foods but not for energy-dense foods. This is consistent with previous studies showing that higher socioeconomic status is associated with higher intake of nutrients required for a healthy diet [29,31]. Lower intake of healthy foods in lower socio-economic groups may be related to cost barriers, measured by income, and individual capacity to make use of public health information, assessed by education level [59,60,61].

Regarding psychological characteristics, dietary restraint also appears to be a strong determinant of dietary intake, particularly in overweight participants. Higher intake of nutrient-dense foods and lower intake of energy-dense foods were observed in high restrainers, in concordance with a previous study [25]. Nearly all overweight and obese participants were former or current dieters, and had higher scores for cognitive restraint than non-overweight participants, thus explaining the stronger influence of dietary restraint on consumption of energy-dense food in this population. In contrast, high uncontrolled eating was associated with high intake of energy-dense foods in all participants, as well as emotional eating in non-overweight participants, in line with previous studies [25,26]. Physical activity was also associated with intake of nutrient-dense foods in both samples, in line with a recent study showing that active adults have higher intake of essential micronutrients [62].

Our findings also showed that men had higher intake of energy-dense and nutrient-dense foods, probably due to their greater need for energy compared to women [63]. In addition, men had greater sensory liking for fat than women, in line with a previous study reporting that women were more likely than men to prefer the taste of nutrient-dense foods [64]. Finally, older individuals had lower fat liking than younger persons, in accordance with a previous study [42] and an experimental work [65], probably due to physiological changes [66].

Interpretation of the present results must take into account characteristics of the cohort. Subjects were volunteers in the NutriNet-Santé cohort and, thus, were probably more concerned about healthy lifestyle and nutrition than the general population. Furthermore, as the collection and analysis of ethnicity characteristics is not allowed in French studies, it could not be considered in the analyses. In addition, only participants who completed questionnaires used in this analysis and who were not lost to follow-up after five years were included, which limits the representativeness of the samples. Thus, caution is needed when interpreting and generalizing the results. In contrast, the prospective design of the study with a five-year follow-up is a strength and enabling us to explore relationships between various direct and distant predictors of weight gain. Individual characteristics and dietary intake were assessed only at baseline; therefore, the cumulative effect of these behaviors on weight change could not be assessed. However, slight changes of dietary intake and physical activity have been shown between baseline and five years later, highlighting that behaviors were unchanging during five years in this population (Supplemental Table S1). Other studies have also shown quite stable lifestyles with slight changes in dietary intake [67,68] showing that during the few years of follow-up, behaviors did not really change in adults. In addition, regarding physical activity, small changes over five years may not appear due to the three large classes of physical activity level assessed by the IPAQ. In addition, fit indices in the “overweight model” were close to the significance level for two of the three criteria; thus, results should be interpreted with caution. Another potential limitation was that data were self-reported by questionnaires and may not have been as accurate as measured data. One study performed on a NutriNet-Santé cohort sample demonstrated the validity of web-based self-reported anthropometric data by comparison with clinical data (*n* = 2513), and showed that the reporting bias was reasonably small [69]. Finally, the main strength of the study was the use of the structural equation modeling approach, which has rarely been used in nutritional epidemiology [70,71]; it enabled modeling observed and latent constructs (unlike that in traditional regression) and simultaneously testing several hypotheses along with the relative contribution of various factors.

## 5. Conclusions

This original study focused on the relative influence of sensory, psychological, socioeconomic, physical activity, and dietary intake on five-year weight change in non-overweight and overweight participants. Our findings confirmed several well-known relationships found in the literature regarding the influence of age, sex, and socio-economic factors on dietary intake, as well as the influence of dietary intake and physical activity on weight change. Specific associations were also found, such as fat liking as a major determinant of dietary intake in both populations. Finally, dietary restraint was a direct predictor of weight gain compared to other determinants, although dietary restraint appeared to be associated with a healthier diet. Further investigation is warranted to understand this complex relationship and to replicate results in other populations. At a clinical level, a sensory based-intervention [72] may be implemented in populations identified as highly restrained in order to improve their eating-related attitudes and behaviors, and help with weight management.

## Figures and Tables

**Table 1:** Baseline characteristics of the population, *n* = 8014, and subsamples according to baseline body mass index (BMI), NutriNet-Santé cohort, France.

	Total		Non-Overweight Participants		Overweight and Obese Participants		
	*n* = 8014		*n* = 5546		*n* = 2468		*p*-Value *
	% or Mean	SD	% or Mean	SD	% or Mean	SD	
*General characteristics*							
Age, year (20–87 years)	51.8	13.1	50.2	13.2	55.3	12.0	<0.0001
Women, %	71.5		77.1		59.0		<0.0001
BMI, kg/m^2^	23.8	4.2	21.7	1.9	28.7	3.8	<0.0001
Relative weight change, %	1.1	6.5	1.5	0.1	0.4	0.16	<0.0001
Educational level, %							<0.0001
Primary	2.8		1.8		5.0		
Secondary	31.4		28.5		37.8		
Undergraduate	29.7		30.6		27.7		
Postgraduate	36.1		39.1		29.5		
Household income per consumption unit, €/month	2377.8	1218.9	2405.9	1218.0	2314.5	1218.7	0.002
Smoking status, %							<0.0001
Never-smoker	49.7		53.0		42.5		
Former smoker	39.3		35.8		47.1		
Current smoker	11.0		11.2		10.4		
Diet to lose weight, %							<0.0001
Never-dieter	22.0		28.5		7.5		
Former dieter	66.8		64.1		72.9		
Current dieter	11.2		7.4		19.6		
Physical activity, %							<0.0001
Low (0–4074 MET/min/week **)	22.9		21.4		26.1		
Moderate (495–5760 MET/min/week)	42.1		43.5		39.0		
High (1800–19,278 MET/min/week)	35.0		35.1		34.9		
Liking for fat-and-salt (0–10 points)	3.9	1.4	3.8	1.4	4.1	1.3	<0.0001
Liking for fat-and-sweets (0–10 points)	3.6	1.8	3.6	1.7	3.7	1.8	0.1
Cognitive restraint (0–100 points)	43.1	20.3	41.0	20.9	47.7	17.9	<0.0001
Uncontrolled eating (0–100 points)	27.7	17.4	25.2	16.2	33.4	18.6	<0.0001
Emotional eating (0–100 points)	36.3	26.8	32.7	25.3	44.6	28.3	<0.0001
Energy, kcal/day	1990.7	517.3	1957.8	496.9	2064.5	553.4	<0.0001
*Food group consumption, g/day*							
Fruits and vegetables	547.1	255.0	552.0	254.8	536.1	255.4	0.01
Meat and processed meat	78.0	56.6	69.6	51.5	97.0	62.7	<0.0001
Fish	47.0	47.8	46.0	46.5	49.3	50.5	0.005
Starchy food	193.4	110.9	187.6	109.0	206.3	113.9	<0.0001
Whole grain products	37.8	52.8	40.0	54.2	32.9	19.4	<0.0001
Cheese	39.9	31.6	38.7	30.6	42.6	33.5	<0.0001
Milk and yogurt	176.4	161.3	172.8	158.8	184.3	166.4	0.006
Salted snacks, appetizers and sauce	23.6	22.6	23.1	21.7	24.7	24.4	0.004
Oil	9.8	9.9	9.9	10.1	9.4	9.4	0.05
Butter and other added fats	13.8	13.4	13.6	13.2	14.3	13.9	0.06
Fatty-sweet products	104.2	85.6	104.6	84.5	103.3	88.2	0.53
Sugar and sugary product	26.3	26.7	27.3	27.1	23.9	25.6	<0.0001
Sweetened beverages	71.1	167.3	64.2	154.5	86.5	191.3	<0.0001
Alcoholic beverages	116.6	168.5	103.8	150.1	145.5	200.8	<0.0001

* *p* values are for comparison between non-overweight and overweight/obese subjects and were determined using Student’s *t*-test or chi-square test as appropriate. ** MET/min/week: Metabolic Equivalent Task/minute/week.

## Supplementary Materials

The Supplementary File is available online at http://www.mdpi.com/2072-6643/9/11/1179/s1.

## References

[1] World Health Organization (2003). Diet, Nutrition and the Prevention of Chronic Diseases. Joint WHO/FAO Expert Consultation.

[2] Kopelman P.G. (2000). Obesity as a medical problem. Nature.

[3] Jebb S.A. (2007). Dietary determinants of obesity. Obes. Rev..

[4] Du H., Feskens E. (2010). Dietary determinants of obesity. Acta Cardiol..

[5] Buckland G., Bach A., Serra-Majem L. (2008). Obesity and the Mediterranean diet: A systematic review of observational and intervention studies. Obes. Rev..

[6] Maskarinec G., Takata Y., Pagano I., Carlin L., Goodman M.T., Le M.L., Nomura A.M., Wilkens L.R., Kolonel L.N. (2006). Trends and dietary determinants of overweight and obesity in a multiethnic population. Obesity (Silver Spring).

[7] Van Dam R.M., Seidell J.C. (2007). Carbohydrate intake and obesity. Eur. J. Clin. Nutr..

[8] Mozaffarian D., Hao T., Rimm E.B., Willett W.C., Hu F.B. (2011). Changes in diet and lifestyle and long-term weight gain in women and men. N. Engl. J. Med..

[9] Lassale C., Galan P., Castetbon K., Peneau S., Mejean C., Hercberg S., Kesse-Guyot E. (2013). Differential association between adherence to nutritional recommendations and body weight status across educational levels: A cross-sectional study 4. Prev. Med..

[10] Fogelholm M., Anderssen S., Gunnarsdottir I., Lahti-Koski M. (2012). Dietary macronutrients and food consumption as determinants of long-term weight change in adult populations: A systematic literature review. Food Nutr. Res..

[11] Jackson C.L., Hu F.B. (2014). Long-term associations of nut consumption with body weight and obesity. Am. J. Clin. Nutr..

[12] Wane S., van Uffelen J.G., Brown W. (2010). Determinants of weight gain in young women: A review of the literature. J. Womens Health (Larchmt).

[13] Akande V.O., Hendriks A.M., Ruiter R.A., Kremers S.P. (2015). Determinants of dietary behavior and physical activity among Canadian Inuit: A systematic review. Int. J. Behav. Nutr. Phys. Act..

[14] Dulloo A.G., Montani J.P. (2015). Pathways from dieting to weight regain, to obesity and to the metabolic syndrome: An overview. Obes. Rev..

[15] Lowe M.R., Doshi S.D., Katterman S.N., Feig E.H. (2013). Dieting and restrained eating as prospective predictors of weight gain. Front. Psychol..

[16] Tian J., Venn A., Otahal P., Gall S. (2015). The association between quitting smoking and weight gain: A systemic review and meta-analysis of prospective cohort studies. Obes. Rev..

[17] Ball K., Crawford D. (2005). Socioeconomic status and weight change in adults: A review. Soc. Sci. Med..

[18] Cox D.N., Hendrie G.A., Carty D. (2016). Sensitivity, hedonics and preferences for basic tastes and fat amongst adults and children of differing weight status: A comprehensive review. Food Qual. Prefer..

[19] Deglaire A., Mejean C., Castetbon K., Kesse-Guyot E., Hercberg S., Schlich P. (2015). Associations between weight status and liking scores for sweet, salt and fat according to the gender in adults (The Nutrinet-Sante study). Eur. J. Clin. Nutr..

[20] Lampure A., Castetbon K., Deglaire A., Schlich P., Peneau S., Hercberg S., Mejean C. (2016). Associations between liking for fat, sweet or salt and obesity risk in French adults: A prospective cohort study. Int. J. Behav. Nutr. Phys. Act..

[21] Mejean C., Deglaire A., Kesse-Guyot E., Hercberg S., Schlich P., Castetbon K. (2014). Association between intake of nutrients and food groups and liking for fat (The Nutrinet-Sante Study). Appetite.

[22] De Lauzon-Guillain B., Basdevant A., Romon M., Karlsson J., Borys J.M., Charles M.A. (2006). Is restrained eating a risk factor for weight gain in a general population?. Am. J. Clin. Nutr..

[23] Chaput J.P., LeBlanc C., Perusse L., Despres J.P., Bouchard C., Tremblay A. (2009). Risk factors for adult overweight and obesity in the Quebec Family Study: Have we been barking up the wrong tree?. Obesity (Silver Spring).

[24] Peneau S., Menard E., Mejean C., Bellisle F., Hercberg S. (2013). Sex and dieting modify the association between emotional eating and weight status. Am. J. Clin. Nutr..

[25] De Lauzon B., Romon M., Deschamps V., Lafay L., Borys J.M., Karlsson J., Ducimetiere P., Charles M.A. (2004). The Three-Factor Eating Questionnaire-R18 is able to distinguish among different eating patterns in a general population. J. Nutr..

[26] Camilleri G.M., Mejean C., Kesse-Guyot E., Andreeva V.A., Bellisle F., Hercberg S., Peneau S. (2014). The Associations between Emotional Eating and Consumption of Energy-Dense Snack Foods Are Modified by Sex and Depressive Symptomatology. J. Nutr..

[27] Sarrafzadegan N., Talaei M., Sadeghi M., Mohammadifard N., Taheri M., Lotfizadeh M., Esmaillzadeh A., Khosravi-Boroujeni H. (2014). Determinants of weight change in a longitudinal study of Iranian adults: Isfahan Cohort Study. Arch. Iran. Med..

[28] Forbes G.B. (1987). Human Body Composition.

[29] Darmon N., Drewnowski A. (2008). Does social class predict diet quality?. Am. J. Clin. Nutr..

[30] Mejean C., Si H.W., Lecossais C., Alles B., Peneau S., Hercberg S., Castetbon K. (2016). Socio-economic indicators are independently associated with intake of animal foods in French adults. Public Health Nutr..

[31] Si Hassen W., Castetbon K., Cardon P., Enaux C., Nicolaou M., Lien N., Terragni L., Holdsworth M., Stronks K., Hercberg S. (2016). Socioeconomic Indicators Are Independently Associated with Nutrient Intake in French Adults: A DEDIPAC Study. Nutrients.

[32] Hercberg S., Castetbon K., Czernichow S., Malon A., Mejean C., Kesse E., Touvier M., Galan P. (2010). The Nutrinet-Sante Study: A web-based prospective study on the relationship between nutrition and health and determinants of dietary patterns and nutritional status. BMC Public Health.

[33] Touvier M., Kesse-Guyot E., Mejean C., Pollet C., Malon A., Castetbon K., Hercberg S. (2011). Comparison between an interactive web-based self-administered 24 h dietary record and an interview by a dietitian for large-scale epidemiological studies. Br. J. Nutr..

[34] World Health Organization (1995). Physical Status: The Use and Interpretation of Anthropometry.

[35] Lassale C., Castetbon K., Laporte F., Camilleri G.M., Deschamps V., Vernay M., Faure P., Hercberg S., Galan P., Kesse-Guyot E. (2015). Validation of a Web-based, self-administered, non-consecutive-day dietary record tool against urinary biomarkers. Br. J. Nutr..

[36] Lassale C., Castetbon K., Laporte F., Deschamps V., Vernay M., Camilleri G.M., Faure P., Hercberg S., Galan P., Kesse-Guyot E. (2016). Correlations between Fruit, Vegetables, Fish, Vitamins, and Fatty Acids Estimated by Web-Based Nonconsecutive Dietary Records and Respective Biomarkers of Nutritional Status. J. Acad. Nutr. Diet..

[37] Hercberg S., Chat-Yung S., Chauliac M. (2008). The French National Nutrition and Health Program: 2001–2006–2010. Int. J. Public Health.

[38] Black A.E. (2000). Critical evaluation of energy intake using the Goldberg cut-off for energy intake:basal metabolic rate. A practical guide to its calculation, use and limitations. Int. J. Obes. Relat. Metab. Disord..

[39] Johansson G., Wikman A., Ahren A.M., Hallmans G., Johansson I. (2001). Underreporting of energy intake in repeated 24-hour recalls related to gender, age, weight status, day of interview, educational level, reported food intake, smoking habits and area of living. Public Health Nutr..

[40] Craig C.L., Marshall A.L., Sjostrom M., Bauman A.E., Booth M.L., Ainsworth B.E., Pratt M., Ekelund U., Yngve A., Sallis J.F. (2003). International physical activity questionnaire: 12-country reliability and validity. Med. Sci. Sports Exerc..

[41] Deglaire A., Mejean C., Castetbon K., Kesse-Guyot E., Urbano C., Hercberg S., Schlich P. (2012). Development of a questionnaire to assay recalled liking for salt, sweet and fat. Food Qual. Prefer..

[42] Lampure A., Deglaire A., Schlich P., Castetbon K., Peneau S., Hercberg S., Mejean C. (2014). Liking for fat is associated with sociodemographic, psychological, lifestyle and health characteristics. Br. J. Nutr..

[43] Stunkard A.J., Messick S. (1985). The three-factor eating questionnaire to measure dietary restraint, disinhibition and hunger. J. Psychosom. Res..

[44] Tholin S., Rasmussen F., Tynelius P., Karlsson J. (2005). Genetic and environmental influences on eating behavior: The Swedish Young Male Twins Study. Am. J. Clin. Nutr..

[45] Christ S.L., Lee D.J., Lam B.L., Zheng D.D. (2014). Structural equation modeling: A framework for ocular and other medical sciences research 90. Ophthalmic Epidemiol..

[46] Joreskog K.G. (1970). A general method for analysis of covariance structures. Biometrika.

[47] Hu L.T., Bentler P.M. (1999). Cutoff criteria for fit indexes in covariance structure analysis: Conventional criteria versus new alternatives. Struct. Equ. Model..

[48] Rosseel Y. (2012). Lavaan: An R package for Structural Equation Modeling. J. Stat. Softw..

[49] Barte J.C., Veldwijk J., Teixeira P.J., Sacks F.M., Bemelmans W.J. (2014). Differences in weight loss across different BMI classes: A meta-analysis of the effects of interventions with diet and exercise. Int. J. Behav. Med..

[50] Smith C.F., Williamson D.A., Bray G.A., Ryan D.H. (1999). Flexible vs. Rigid dieting strategies: Relationship with adverse behavioral outcomes. Appetite.

[51] Benard M., Camilleri G.M., Etile F., Mejean C., Bellisle F., Reach G., Hercberg S., Peneau S. (2017). Association between Impulsivity and Weight Status in a General Population. Nutrients.

[52] Schaumberg K., Anderson D.A., Anderson L.M., Reilly E.E., Gorrell S. (2016). Dietary restraint: What’s the harm? A review of the relationship between dietary restraint, weight trajectory and the development of eating pathology. Clin. Obes..

[53] Lowe M.R., Feig E.H., Winter S.R., Stice E. (2015). Short-term variability in body weight predicts long-term weight gain. Am. J. Clin. Nutr..

[54] Guerra F., Stringhini S., Vollenweider P., Waeber G., Marques-Vidal P. (2015). Socio-demographic and behavioural determinants of weight gain in the Swiss population. BMC Public Health.

[55] Hughes V.A., Frontera W.R., Roubenoff R., Evans W.J., Singh M.A. (2002). Longitudinal changes in body composition in older men and women: Role of body weight change and physical activity. Am. J. Clin. Nutr..

[56] Glanz K., Basil M., Maibach E., Goldberg J., Snyder D. (1998). Why Americans eat what they do: Taste, nutrition, cost, convenience, and weight control concerns as influences on food consumption. J. Am. Diet. Assoc..

[57] Drewnowski A., Hann C. (1999). Food preferences and reported frequencies of food consumption as predictors of current diet in young women. Am. J. Clin. Nutr..

[58] Nagata C., Sugiyama C., Shimizu H. (1998). Nutrient intakes in realtion to style of breakfast and taste preferences. J. Epidemiol..

[59] Reicks M., Randall J.L., Haynes B.J. (1994). Factors affecting consumption of fruits and vegetables by low-income families. J. Am. Diet. Assoc..

[60] Galobardes B., Morabia A., Bernstein M.S. (2001). Diet and socioeconomic position: Does the use of different indicators matter?. Int. J. Epidemiol..

[61] Cox D.N., Anderson A.S., McKellar S., Reynolds J., Lean M.E.J., Mela D.J. (1996). Vegetables and fruits: Barriers and opportunities for greater consumption. Nutr. Food Sci..

[62] Yan Y., Drenowatz C., Hand G.A., Shook R.P., Hurley T.G., Hebert J.R., Blair S.N. (2016). Is nutrient intake associated with physical activity levels in healthy young adults?. Public Health Nutr..

[63] Mauvais-Jarvis F. (2015). Sex differences in metabolic homeostasis, diabetes, and obesity. Biol. Sex Differ..

[64] Turrell G. (1997). Determinants of gender differences in dietary behavior. Nutr. Res..

[65] Warwick Z.S., Schiffman S.S. (1990). Sensory evaluations of fat-sucrose and fat-salt mixtures: Relationship to age and weight status 4. Physiol. Behav..

[66] Drewnowski A., Shultz J.M. (2001). Impact of aging on eating behaviors, food choices, nutrition, and health status. J. Nutr. Health Aging.

[67] Kimokoti R.W., Newby P.K., Gona P., Zhu L., Campbell W.R., D’Agostino R.B., Millen B.E. (2012). Stability of the Framingham Nutritional Risk Score and its component nutrients over 8 years: The Framingham Nutrition Studies 4. Eur. J. Clin. Nutr..

[68] Nanri A., Shimazu T., Ishihara J., Takachi R., Mizoue T., Inoue M., Tsugane S. (2012). Reproducibility and validity of dietary patterns assessed by a food frequency questionnaire used in the 5-year follow-up survey of the Japan Public Health Center-Based Prospective Study 20. J. Epidemiol..

[69] Lassale C., Peneau S., Touvier M., Julia C., Galan P., Hercberg S., Kesse-Guyot E. (2013). Validity of web-based self-reported weight and height: Results of the Nutrinet-Sante study. J. Med. Internet Res..

[70] Yuan W.L., Rigal N., Monnery-Patris S., Chabanet C., Forhan A., Charles M.A., de Lauzon-Guillain B. (2016). Early determinants of food liking among 5y-old children: A longitudinal study from the EDEN mother-child cohort. Int. J. Behav. Nutr. Phys. Act..

[71] Kesse-Guyot E., Andreeva V.A., Lassale C., Hercberg S., Galan P. (2014). Clustering of midlife lifestyle behaviors and subsequent cognitive function: A longitudinal study. Am. J. Public Health.

[72] Gravel K., Deslauriers A., Watiez M., Dumont M., Dufour Bouchard A.A., Provencher V. (2014). Sensory-based nutrition pilot intervention for women. J. Acad. Nutr. Diet..

