# Supplementary material for: Relative Influence of Socioeconomic, Psychological and Sensory Characteristics, Physical Activity and Diet on 5-Year Weight Gain in French Adults
Source: Nutrients. 2017 Oct 28;9(11):1179. doi: 10.3390/nu9111179 (PMC5707651; doi:10.3390/nu9111179)
Supplement: Supplementary file 1 [file nutrients-09-01179-s001.pdf]

Supplemental Table S1. Dietary intake of food groups and physical activity at baseline and at five-year follow-up, NutriNet-Santé cohort.

	Baseline data<br>% or mean ±SD	Follow-up data<br>% or mean ±SD	*p*-value
Food group consumption, g/d			
Fruits and vegetables	547.1 ± 255.0	542.1 ± 252.5	<0.0001
Meat and processed meat	78.0 ± 56.6	73.8 ± 54.8	0.0002
Fish	47.0 ± 47.8	41.4 ± 45.8	<0.0001
Starchy foods	193.4 ± 110.9	184.4 ± 102.0	<0.0001
Whole grain products	37.8 ± 52.8	38.9 ± 52.0	0.19
Cheese	39.9 ± 31.6	40.0 ± 30.8	0.0009
Milk and yogurts	176.4 ± 161.3	157.3 ± 151.6	<0.0001
Salted snacks, appetizers and sauces	23.6 ± 22.6	25.1 ± 22.6	0.01
Oil	9.8 ± 9.9	9.9 ± 9.9	0.87
Butter and other added fats	13.8 ± 13.4	14.5 ± 13.5	0.85
Fatty-sweet products	104.2 ± 85.6	99.1 ± 81.2	0.002
Sugar and sugary products	27.3 ± 27.1	27.7 ± 26.6	0.17
Sweetened soft drinks	71.1 ± 167.3	66.2 ± 155.4	<0.0001
Alcoholic beverages	116.6 ± 168.5	113.8 ± 154.1	<0.0001
Energy, kcal/d	1990.7 ± 517.3	1963.1 ± 486.9	<0.0001
Physical activity, %			0.0002
Low	22.9	21.4	
Moderate	42.1	41.7	
High	35.0	36.9	

Baseline data n = 8014, five-year follow-up data n = 4103 for dietary intake, and n = 7732 for physical activity.

p-values are for comparison between baseline and five-year follow-up data and were determined using paired Student's *t*-test or Bowker's test of symmetry, as appropriate.

Supplemental Table S2. Correlation of baseline characteristics of included participants, NutriNet-Santé cohort, n = 8014.

	Age	Educational level	Income per consumption unit	Smoking status	Physical activity	Dieting to lose weight	Liking for fat-and-salt	Liking for fat-and-sweet	Cognitive restraint	Emotional eating	Uncontrolled eating
Age	1										
Educational level	–0.26090	1									
	<0.0001										
Income per consumption unit	0.22735	0.30722	1								
	<0.0001	<0.0001									
Smoking status	0.10207	–0.06315	0.00148	1							
	<0.0001	<0.0001	0.8946								
Physical activity	0.22217	–0.10409	0.00985	0.01053	1						
	<0.0001	<0.0001	0.3779	0.3459							
Dieting to lose weight	–0.01483	–0.04317	–0.00740	0.05433	–0.03445	1					
	0.1845	0.0001	0.5078	<0.0001	0.0020						
Liking for fat-and-salt	–0.21749	0.04666	–0.08523	0.01848	–0.17482	0.02598	1				
	<0.0001	<0.0001	<0.0001	0.0980	<0.0001	0.0200					
Liking for fat-and-sweet	–0.30263	0.05075	–0.12542	–0.03097	–0.14578	0.05164	0.54366	1			
	<0.0001	<0.0001	<0.0001	0.0056	<0.0001	<0.0001	<0.0001				
Cognitive restraint	0.11034	–0.05804	0.03785	0.01900	0.03625	0.47958	–0.10797	–0.03280	1		
	<0.0001	<0.0001	0.0007	0.0890	0.0012	<0.0001	<0.0001	0.0033			
Emotional eating	–0.18662	0.00767	–0.08668	–0.06594	–0.10530	0.32598	0.09024	0.19157	0.30987	1	
	<0.0001	0.4927	<0.0001	<0.0001	<0.0001	<0.0001	<0.0001	<0.0001	<0.0001		

Uncontrolled eating	–0.15916	–0.00850	–0.07990	0.01206	–0.07418	0.24648	0.21751	0.23632	0.24010	0.59782	1
	<0.0001	0.4468	<0.0001	0.2804	<0.0001	<0.0001	<0.0001	<0.0001	<0.0001	<0.0001	

Correlation coefficient and *p*-values of Pearson correlation for age, liking for fat-and-salt, liking for fat-and-sweet, cognitive restraint, emotional eating and uncontrolled eating scores, and Spearman correlation for educational level, income, smoking status, physical activity, and dieting to lose weight.

Supplemental Table S3. Comparison of baseline characteristics between included and excluded participants which have available anthropometric data at baseline and at five-year follow-up, n = 14,620, NutriNet-Santé cohort, May 2010.

	**Included participants** n = 8014	**Excluded participants** N = 6606	*p*-value
General characteristics			
Age, y	51.8 ± 13.1	50.0 ± 13.6	<0.0001
Women, %	71.5	73.3	0.019
BMI, kg/m²	23.8 ± 4.2	24.0 ± 4.4	0.056
Educational level, % [1]			<0.0001
Elementary school	2.8	3.6	
Secondary school	31.4	35.8	
College graduate	29.7	29.9	
Advanced degree	36.1	30.7	
Income per consumption unit, € [1]	2377.8 ± 1218.9	2241.8 ± 1223.7	<0.0001
Smoking status, % [1]			<0.0001
Never-smoker	49.7	49.6	
Former smoker	39.3	37.5	
Current smoker	11.0	12.9	

p-values are for comparison between included and excluded participants and were determined using Student's *t*-test or chi-square test as appropriate.

[1] For 14,190 individuals.

Supplemental Table S4. Comparison of baseline characteristics between included and excluded participants of the analyses, NutriNet-Santé cohort, n = 49,818, May 2010.

	**Included participants**<br>n = 8014	**Excluded participants**<br>n = 41,804	*p*-value
General characteristics			
Age, y	51.8 ± 13.1	43.9 ± 14.5	<0.0001
Women, %	71.5	77.8	<0.0001
BMI, kg/m² [1]	23.8 ± 4.2	23.9 ± 4.7	0.24
Educational level, % [2]			<0.0001
Elementary school	2.8	3.1	
Secondary school	31.4	34.9	
College graduate	29.7	30.3	
Advanced degree	36.1	31.7	
Income per consumption unit, € [2]	2377.8 ± 1218.9	2098.1 ± 1186.1	<0.0001
Smoking status, % [2]			<0.0001
Never-smoker	49.7	49.1	
Former smoker	39.3	34.3	
Current smoker	11.0	16.6	

p-values are for comparison between included and excluded participants and were determined using Student's *t*-test or chi-square test as appropriate.

[1] For 31,407 individuals.
